# Bioremediation of Direct Blue 14 and Extracellular Ligninolytic Enzyme Production by White Rot Fungi: *Pleurotus* Spp.

**DOI:** 10.1155/2013/180156

**Published:** 2013-06-11

**Authors:** M. P. Singh, S. K. Vishwakarma, A. K. Srivastava

**Affiliations:** Department of Biotechnology, VBS Purvanchal University, Jaunpur, UP 222001, India

## Abstract

In the present investigation, four species of white rot fungi (*Pleurotus*), that is, *P. flabellatus, P. florida, P. ostreatus* and *P. sajor-caju* were used for decolorization of direct blue 14 (DB14). Among all four species of *Pleurotus*, *P. flabellatus* showed the fastest decolorization in petri plates on different concentration, that is, 200 mg/L, 400 mg/L, and 600 mg/L. All these four species were also evaluated for extracellular ligninolytic enzymes (laccase and manganese peroxidase) production and it was observed that the twelve days old culture of *P. flabellatus* showed the maximum enzymatic activity, that is, 915.7 U/mL and 769.2 U/mL of laccase and manganese peroxidase, respectively. Other three *Pleurotus* species took more time for dye decolorization and exhibited less enzymatic activities. The rate of decolorization of DB14 dye solution (20 mg/L) by crude enzymes isolated from *P. flabellatus* was very fast, and it was observed that up to 90.39% dye solution was decolorized in 6 hrs of incubation.

## 1. Introduction 

Azo dyes are the largest class of synthetic dyes used for textile dyeing, paper printing, and other industrial applications. During the dyeing process, 5–20% of the used dyestuffs are released into the processed water [1, 2]. It was estimated that only textile industries alone generate about 4500 million kiloliters of wastewaters annually. As these dyes are synthetic, they contain toxic content in the effluents because partial degradation of these dyes results in aromatic amines that are toxic to aquatic life and mutagenic and carcinogenic to humans [3]. There is a big challenge to remove dye content from industrial effluents. Various physical, chemical, and biological methods have been proposed [4] among these, microbiological and enzymatic decomposition have received much attention in recent years [5–7]. Although, many reports are available for dye decolorization and degradation by bacteria, the reductive products of dye degradation are generally aromatic amines which are potentially hazardous to living organisms [8, 9] and due to larger size of dyes, bacteria are unable to degrade these dyes efficiently. 

White rot fungi degrade lignin because they secrete oxidoreductases including lignin peroxidase (1,2-bis (3,4-dimethoxyphenyl) propene-1,3-diol:hydrogen-peroxide-Lip EC 1.1.1.14), manganese peroxidase (Mn(II):hydrogen-peroxide oxidoreductase EC 1.11.1.13), and laccase (benzenediol:oxygen reductase EC 1.10.3.2). These enzymes oxidise in a nonspecific way both phenolic and nonphenolic lignin derivatives and thus are promising candidates for the degradation of environmental pollutants, for example, phenols, anilines, dyes, lignocelluloses [10–14] and highly recalcitrant compounds such as polychlorinated biphenyls (PCBs) and polycyclic aromatic hydrocarbons (PAHs) [15]. Biodegradation of several persistent compounds such as dyes, pesticides and lignin derivatives has been attributed to the oxidative enzymes, especially laccase [16, 17]. The present research work is undertaken to evaluate the potentialities of *Pleurotus *species for dye decolorization.

## 2. Materials and Methods

### 2.1. Cultures and Their Maintenance

The pure cultures of *Pleurotus* species, that is, *P. flabellatus*, *P. florida*, *P. ostreatus,* and* P. sajor-caju* were obtained from Directorate of Mushroom Research, Solan (Himachal Pradesh), India. Throughout the study, cultures were maintained on MEA (Malt extract agar) medium at 28°C and subcultured at the regular interval of three weeks. 

### 2.2. Screening of Species for Dye Decolorization

DB14 (direct blue 14) was used as reference dye for group of azo dye. Inoculums of four *Pleurotus* spp. (*P. flabellatus*, *P. florida*, *P. ostreatus* and* P. sajor-caju*) were inoculated on MEA plate containing 200 mg/L, 400 mg/L, and 600 mg/L of DB14. These plates were incubated on 28 ± 2°C. 

### 2.3. Production of Enzymes

The medium for enzyme production contained 2% wheat bran and 2.5% malt extract, and the pH was adjusted to 6.0 by using NaOH or HCl. Incubation was carried out at 28°C in biological oxygen demand BOD incubator in cotton-plugged 250 mL Erlenmeyer flasks containing 50 mL of media. Flasks were inoculated with 1 cm^2^ agar pieces from actively growing fungus on malt extract agar plate. 

### 2.4. Extraction of Extracellular Enzymes

Samples of substrate were collected at regular interval of 3 days and extracted in phosphate buffer (pH 6.0) for ligninolytic enzymes. Filtrate of extraction was used for enzyme assay.

### 2.5. Enzyme Assay

Laccase activity was determined *via *the oxidation of o-methoxyphenol catechol monomethylether (guaiacol) as substrate. The reaction mixture contained 1 mL of 1 mM guaiacol in 0.1 M sodium phosphate buffer (pH 6.0) and 1 mL of crude enzyme solution was incubated at 30°C for 10 min. The oxidation was followed by the increase in absorbance at 495 nm. One activity unit was defined as 1 *μ*mol of guaiacol oxidised per minute [18].

Manganese peroxidase (MnP) activity was determined using guaiacol as substrate. The reaction mixture contained 0.2 mL of 0.5 M Na-tartrate buffer (pH 5.0), 0.1 mL of 1 mM MnSO_4_, 0.1 mL of 1 mM H_2_O_2_, 0.25 mL of 1 mM guaiacol and 0.3 mL of crude enzymes. The oxidation of substrate at 30°C was followed spectrophotometrically at (*A*_465_) [19]. 

### 2.6. Decolorization of Synthetic Dye by Crude Enzyme Solution

Decolorization of the azo dye direct blue 14 was monitored in a mixture containing 1.4 mL DB14 solution (20 mg L^−1^), 0.2 mL 0.5 M sodium acetate buffer pH 3.5, 0.2 mL crude enzyme, 0.2 mL of deionised water, 0.1 M H_2_O_2_, 0.1 M MnSO_4_ or 5.5 mM ABTS solutions, at 25 ± 2°C [20].

Visible spectra were recorded with a UV-visible (Elico-SL191) spectrophotometer at *λ* = 595 nm. The rate of decolorization was expressed as the percentage decrease in absorbance at the peak wavelength. Control tests were conducted with crude enzyme replaced by deionised water. Experiments were performed in triplicate, and results were expressed as the mean values.

## 3. Results and Discussion 

**Figure 1:**
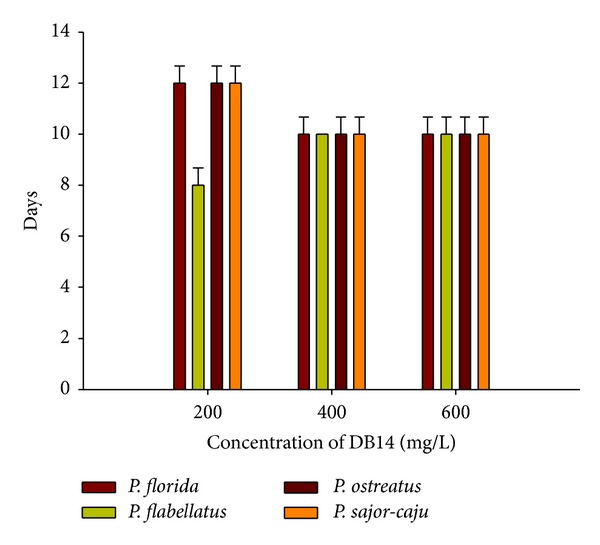
Decolorization of DB14 by *Pleurotus* spp.

All four species of *Pleurotus* selected in the present work showed different rate of decolorization of DB14. Among the four species of *Pleurotus, P. flabellatus *showed the fastest decolorization of DB14 followed by* P. florida*, *P. sajor-caju, *and* P. ostreatus *on 200 mg/L of DB14 (Figure 1). *P. flabellatus *completely decolorized DB14 within 8 days. *P. flabellatus *effectively decolorized the dye at the concentration of 400 mg/L and also at 600 mg/L. At 400 mg/L concentration complete decolorization occurred in 10 days and other species took almost the similar time, but the rate of decolorization and mycellial growth were better in case of *P. flabellatus*. Same results were observed at 600 mg/L concentration. The degrees of decolorization of different dyes such as malachite green, indigo carmine, xylidine ponceau, Bismarck brown and methyl orange using the white rot fungus *P. ostreatus* were previously evaluated by Neelamegam *et al.* [21]. This study demonstrated the potentialities of white rot fungi in bioremediation of dye contaminated ecosystems.

**Figure 2:**
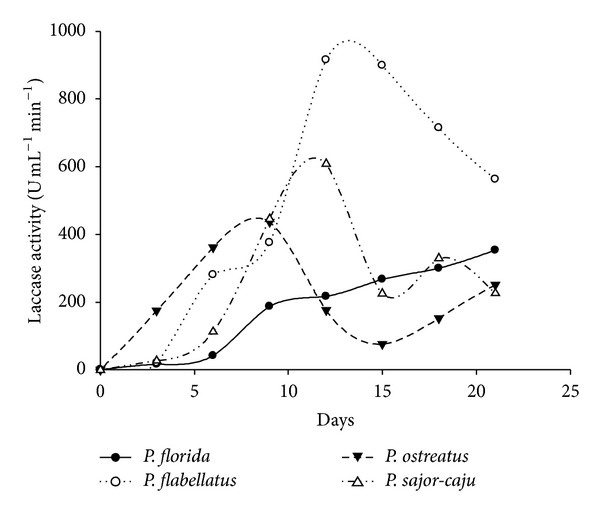
Laccase production by different *Pleurotus* species in submerged condition.

**Figure 3:**
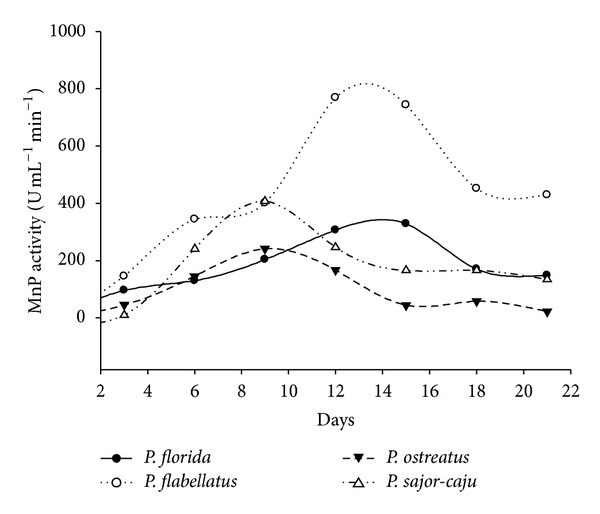
MnP production by different *Pleurotus* species in submerged condition.

The time course of laccase and MnP activity was followed in the wheat bran supplemented liquid media over a period of 21 days. Initially, it was observed that amongst the four species of *Pleurotus, P. flabellatus *showed the highest laccase activities on all days evaluated, reaching maximum levels of 915.7 U/mL in 12 days of culture of *P. flabellatus *on wheat bran containing media. This was followed by *P. sajor-caju *which showed maximum laccase activity (608.6 U/mL) in 12 days. Subsequently, *P. ostreatus *and *P. florida *showed maximum laccase activity, that is, 436.3 U/mL and 353.3 U/mL in 9 days and 21 days, respectively (Figure 2). MnP activities were detected at levels of up to 769.2 U/mL by *P. flabellatus *in 12 days old culture followed by *P. Sajor-caju*, *P. ostreatus, *and *P. florida* that is, 407.4 U/mL, 241.8 U/mL, and 329 U/mL on 9, 9, and 15 days old culture, respectively (Figure 3).

Every species showed maximum enzymatic activity on the 9th day or after the 9th day of incubation, which might be due to the occurrence of initial lag phase when species try to establish it in new medium. When cultures are established in the culture medium, they enter into log phase, and metabolically this is the most active phase where species show maximum enzymatic activity. Cereal bran was reported to increase ligninolytic enzyme production of the white rot fungi *Coriolopsis gallica *and *Bjerkandera adusta *[22]. In the beginning of the experiment, on day 3, different *Pleurotus *species showed low enzymatic activities. This was followed by sharp increase up to 12 days in *P. flabellatus *and *P. sajor-caju, *whereas in case *P. ostreatus *and *P. florida *it took 9 and 21 days for laccase. *P. flabellatus *and *P. florida, *showed maximum MnP activities in 12 days whereas *P. ostreatus* and *P. sajor-caju* showed the maximum MnP activities in 9 days, respectively. Laccase and MnP both are oxidative enzyme and have broad range of substrate specificity. 

**Figure 4:**
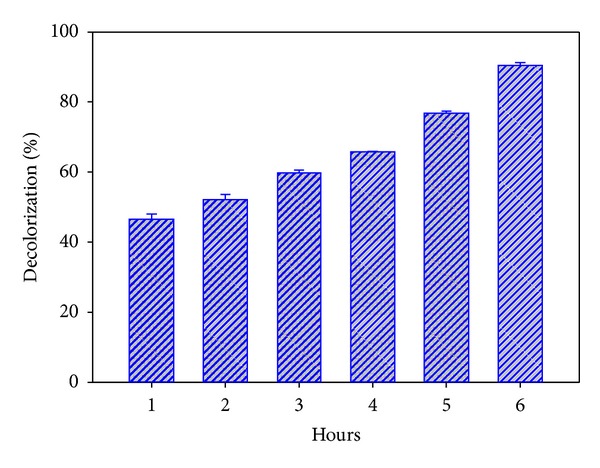
Decolorization of DB14 by crude extracellular enzymes of *P. flabellatus*.

Several authors have discussed the role of lignicolous fungal enzymes in the decolorization of dyes [14, 23, 24]. Crude enzymes obtained from cultures of *P. flabellatus* on wheat bran containing malt extract media were tested for decolorization of DB14.  Figure 4 shows the percent decolorization during various periods. The decolorization of the dye using crude enzyme was 46.49% in the first hour, in present investigation. 90.39% DB14 decolorization was reported in 6 hrs at room temperature (30°C).

The laccase [16] and MnP [25] play a major role in complete oxidation of DB14. It was observed that laccase and peroxidases can act as starters of a chain reaction which leads to dye degradation by generating highly active free radicals (e.g., Mn^3+^, lipid, hydroxyl, and peroxy-radicals) [26, 27]. According to Meyer [28], because of the structural variety of azo dyes, they are not uniformly susceptible to biodegradation. It was demonstrated that substituent groups such as nitro and sulpho are frequently recalcitrant to biodegradation, whereas 2-methyl, 2-methoxy, 2,6-dimethyl and 2,6-dimethoxy-substituted 4-(4-sulfophenylazo)-phenol were preferred for azo-dye degradation by peroxidase from *Streptomyces *spp. and *Phanerochaete chrysosporium* [29]. The breaking down of the dye into smaller fragments, including the breakage of the azo bond, can lead to a decrease in the absorbance of the visible spectra and in a colorless solution [30]. 

## 4. Conclusion

 The present investigation suggests that the white rot fungus *Pleurotus flabellatus* can be used in bioremediation of dye-contaminated ecosystems. This is because of the presence of powerful enzymatic machinery which can effectively degrade the recalcitrant and toxic dyes. Out of four species investigated, *P. flabellatus* showed the maximum enzymatic activity. Extracellular enzymes extracted from 12 days old culture of *P. flabellatus* decolorized the DB14 to maximum extent. For better enzyme production the wheat bran can be used as supplement in liquid media. Decolorized effluent after enzymatic treatment can be reused by industries and also in agriculture.

## Abbreviations

DB14: Direct blue 14
MnP: Manganese peroxidase
hrs: Hours.
